# Environmental temperature variation influences fitness trade-offs and tolerance in a fish-tapeworm association

**DOI:** 10.1186/s13071-017-2192-7

**Published:** 2017-06-02

**Authors:** Frederik Franke, Sophie A. O. Armitage, Megan A. M. Kutzer, Joachim Kurtz, Jörn P. Scharsack

**Affiliations:** Institute for Evolution and Biodiversity, University of Münster, Hüfferstrasse 1, 48149 Münster, Germany

**Keywords:** Host-parasite interaction, Fitness, Tolerance, Environment, Temperature, *Gasterosteus aculeatus*, *Schistocephalus solidus*

## Abstract

**Background:**

Increasing temperatures are predicted to strongly impact host-parasite interactions, but empirical tests are rare. Host species that are naturally exposed to a broad temperature spectrum offer the possibility to investigate the effects of elevated temperatures on hosts and parasites. Using three-spined sticklebacks, *Gasterosteus aculeatus* L., and tapeworms, *Schistocephalus solidus* (Müller, 1776), originating from a cold and a warm water site of a volcanic lake, we subjected sympatric and allopatric host-parasite combinations to cold and warm conditions in a fully crossed design. We predicted that warm temperatures would promote the development of the parasites, while the hosts might benefit from cooler temperatures. We further expected adaptations to the local temperature and mutual adaptations of local host-parasite pairs.

**Results:**

Overall, *S. solidus* parasites grew faster at warm temperatures and stickleback hosts at cold temperatures. On a finer scale, we observed that parasites were able to exploit their hosts more efficiently at the parasite’s temperature of origin. In contrast, host tolerance towards parasite infection was higher when sticklebacks were infected with parasites at the parasite’s ‘foreign’ temperature. Cold-origin sticklebacks tended to grow faster and parasite infection induced a stronger immune response.

**Conclusions:**

Our results suggest that increasing environmental temperatures promote the parasite rather than the host and that host tolerance is dependent on the interaction between parasite infection and temperature. Sticklebacks might use tolerance mechanisms towards parasite infection in combination with their high plasticity towards temperature changes to cope with increasing parasite infection pressures and rising temperatures.

**Electronic supplementary material:**

The online version of this article (doi:10.1186/s13071-017-2192-7) contains supplementary material, which is available to authorized users.

## Background

Both hosts and parasites are under selection pressure to optimise their fitness in response to their local host or parasite partner and at the same time to the local environmental conditions. Recent studies suggest that host-parasite dynamics depend on interactions with environmental factors [1–3], which eventually leads to local adaptation as a consequence of their coevolutionary arms race [4, 5]. Accordingly, changing environments interfere with host-parasite dynamics and a central question is, how are host and parasite fitness influenced by environmental variation? Selection pressures might force hosts and parasites to adapt to local environmental conditions, but might also trigger the ability to respond plastically to changing environments. Here we elucidate the interplay between hosts, parasites and the environment, by comparing sympatric and allopatric host-parasite combinations, whilst at the same time manipulating experimental temperature, a highly influential environmental condition, particularly for ectothermic hosts.

We used the three-spined stickleback, *Gasterosteus aculeatus* L., and its macroparasite tapeworm *Schistocephalus solidus* (Müller, 1776) originating from a cold and a warm site of the Icelandic volcanic lake Mývatn. Millet et al. [6] investigated the genetic structure of the stickleback population of Lake Mývatn and demonstrated that the sticklebacks from their eleven sampling sites (including cold and warm fed sites) showed little genetic variation, but they exhibited significant phenotypic differences. Interestingly, a higher prevalence (26%) of *S. solidus* was detected in sticklebacks from warm-water sites of Lake Mývatn, compared to a lower prevalence (7%) in sticklebacks from cold-water sites of the lake [7]. This could have been caused by biotic factors, e.g. the abundance of copepods, the first intermediate host of *S. solidus*, but might also be a direct effect of temperature.

The cestode *S. solidus* has a complex life-cycle with copepods as the first host and three-spined sticklebacks as the obligatory and specific second intermediate host. *S. solidus* acquires most of its resources from the stickleback resulting in substantially decreased host reproductive ability, e.g. by preventing females from spawning [8–12], before it manipulates the stickleback’s behaviour to increase transmission to its final host, a piscivorous bird [13, 14]. The weight of the mature *S. solidus* is positively correlated with parasite fecundity and is therefore a well suited fitness correlate [15, 16]. Given the severe loss of fitness for infected stickleback, the selection pressure imposed by *S. solidus* must be high. It has been suggested that sticklebacks in populations with high infection pressure become locally adapted and are more resistant, i.e. have lower infection rates and more efficiently constrain *S. solidus* growth [17, 18]. On the other hand, *S. solidus* seems to adapt to the increased resistance of local hosts and become more virulent, i.e. increased infection success and host exploitation rates [17, 18].

As an alternative to investment in resistance measures to depress parasite growth, hosts may also use a tolerance strategy, allowing them to maximise fitness whilst being infected. Tolerance is the ability of hosts to limit the damage caused by a given parasite load [19]. Tolerance is measured at the population level by plotting individual host fitness against individual parasite loads, the reaction norm giving the relationship between the two parameters describes tolerance. For example, a population with a positive or shallow negative reaction norm is more tolerant, i.e. better at minimising the negative fitness effect of an increasing infection intensity, than a population with a steeper negative slope [19, 20]. A number of studies have shown that hosts use tolerance to mitigate fitness losses during infections with microparasites [19, 21–24]. Given that macroparasites can also dramatically reduce host fitness [12, 25, 26], tolerance might be expected to be an important host strategy [27]. Indeed, in an unmanaged Soay sheep population, individuals that were more tolerant to gastrointestinal nematodes, had higher lifetime breeding success [28]. In our study, we use stickleback growth rate as a measure for health tolerance and gonad weight was employed to estimate fecundity tolerance. Both parameters are hypothesised to be affected by the growing *S. solidus* since the parasite drains substantial amounts of nutrients from its host [8, 10, 11]. Although local adaptation for resistance has been shown, evidence of local adaptation for tolerance has not been found to date [21, 22, 24]. In a mesocosm experiment, lake and stream stickleback ecotypes were similarly non-tolerant to an ectoparasite (*Gyrodactylus* sp.), but elevated nutrient load tended to increase tolerance of stream sticklebacks [29], which might suggest that tolerance varies according to environmental conditions.

Adaptation of sticklebacks to local temperatures was suggested by Dittmar et al. [30], who observed stronger immunological disorders and higher mortality in F1 sticklebacks from a brook compared to F1 sticklebacks from a pond during experimental exposure to a heat wave of up to 28 °C. Furthermore, studies with three-spined sticklebacks from a marine origin, which were adapted to cold and warm conditions in the laboratory for one generation, illustrated that cold adapted sticklebacks had offspring with faster growth compared to warm adapted sticklebacks, which suggests considerable transgenerational plasticity in stickleback temperature adaptation [31–33].

It is predicted that increasing temperatures will significantly alter host-parasite interactions, especially in multi-host parasite life-cycles, since the addition of every further host/parasite larval stage increases the possibility of responses [34]. Temperature affects host-parasite interactions through various pathways, e.g. it might alter host food consumption and thereby change the risk of becoming exposed to trophically transmitted parasites [35]. Furthermore, temperature plays a key role in determining the efficacy of the immune system - the host’s most important physiological barrier against parasitation [30, 36, 37]. In this respect it has been shown that increasing temperature coincided with a higher output of trematode cercariae [38–40] and increased infectivity of metacercariae, while the survival of amphipod hosts decreased [41]. Other studies have observed faster life-cycle completion rates at warmer temperatures in nematodes [42] and acanthocephalans [43], which increases the parasite infection pressure on their hosts. A few lines of evidence suggest that higher temperatures can be detrimental to infected sticklebacks, but beneficial to the parasites; for example, an enclosure experiment during the 2003 European heat wave found that moribund sticklebacks had higher parasite burdens compared to survivors [44]; sticklebacks showed lower growth rates and higher mortality after infection with *Vibrio* bacteria at 21 °C compared to 17 °C [45], and *S. solidus* had a faster growth rate at 20 °C compared to 15 °C [46].

In the present study, we used the offspring of sticklebacks and *S. solidus* collected from warm and cold sites in Lake Mývatn to investigate potential mutual adaptations of local host-parasite pairs and their adaptations to the local temperature regimes. We exposed sticklebacks from both sites to sympatric and allopatric *S. solidus* and to a cold and a warm experimental temperature, in a fully crossed experimental design. We analysed host immune traits as well as host and parasite body condition parameters, which served as fitness estimates. We predicted that parasites might gain higher fitness at elevated temperatures, while the stickleback hosts might benefit from lower temperatures. We further hypothesised that both hosts and parasites would be adapted to their local temperature regimes, i.e. that cold-origin hosts and parasites would perform better under cold conditions compared to warm conditions and vice versa. Further, if local adaptation exists, hosts and parasites might exhibit mutual adaptations to the genotype of their local counterparts.

## Methods

### Study lake and sampling

The sticklebacks and parasites originated from Lake Mývatn in northern Iceland, which was formed by volcanic eruptions 2,300 years ago (about 2,300 stickleback generations), and is fed by cold (around 5 °C), but also warm (up to 30 °C) groundwater inputs [7]. Sticklebacks in breeding condition and *S. solidus* parasitized sticklebacks were collected with minnow traps at a cold (65°39′13.38″N, 16°57′45.27″W) and a warm (65°37′41.66″N, 16°55′16.97″W) water site (linear distance 3.4 km) in June 2014. The sperm of one male stickleback were used to in vitro fertilize [47] the egg clutch of a single female stickleback and the fertilized clutches (offspring from one clutch are below referred to as a ‘family’) were then transferred to tubes with 40 ml aerated tap water. The body cavities of infected sticklebacks were opened, the parasites were removed aseptically, weighed and transferred to individual tubes with 10 ml cell culture medium (MEM, Gibco, Thermo Fisher Scientific, USA). *Schistocephalus solidus* larvae and fertilized stickleback egg clutches were transported to our laboratory in Münster, Germany. At each sampling site the water temperature was recorded from June 2014 for twelve months (HOBO Water Temp Pro v2, Onset, USA). The average temperature at the cold sampling site was 4.8 °C (min: 0 °C, max: 18.8 °C), and 18.4 °C (min: 10.5 °C, max: 23.3 °C) at the warm site (see Additional file 1: Figure S1 for annual temperature fluctuations).

### Animal husbandry

After initial feeding with *Artemia salina* nauplii and frozen plankton, the sticklebacks were fed ad libitum with chironomids and kept separated by families in 14 l tanks with recirculating tap water at 16 °C and a light/dark cycle of 14/10 h. For egg production, parasites were size matched to increase the probability of outcrossing [48] and bred in vitro as described previously [49, 50]. Briefly, parasite eggs were washed and stored at 4 °C in the dark. For larval development, *S. solidus* eggs were incubated for 3 weeks at 20 °C in the dark. To initiate hatching, eggs were illuminated for 3 h followed by 8 h darkness overnight, and at least 2 h illumination the next morning. Hatched coracidia were transferred singly to wells of a 24-well plate, with one copepod (*Macrocyclops albidus* (Jurine)) each in 2 ml tap water. After 2 weeks, the copepods were checked under a microscope for the presence of *S. solidus* larvae. Collection, transport of and experimentation with animals in the present study was done in accordance with the local veterinary and animal welfare authorities under the project number 87-51.04.2010.A297.

### Experimental design

**Fig. 1:**
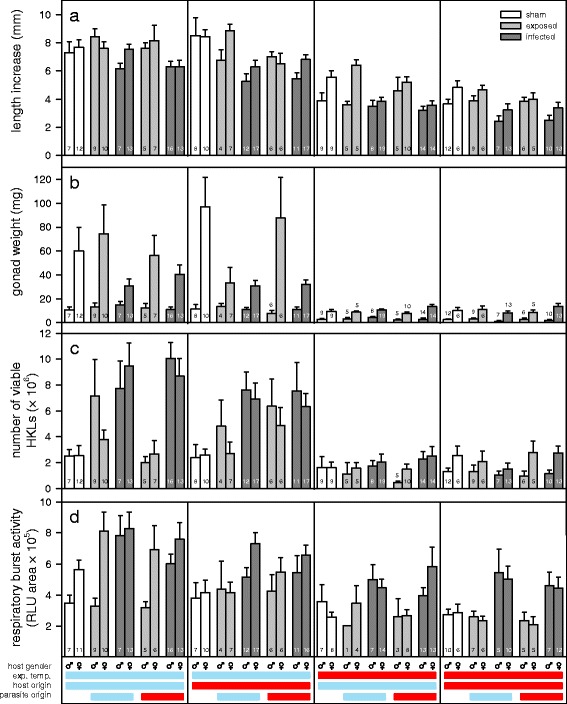
Host responses to temperature variation and *S. solidus* infection. **a** Host length increase. **b** Gonad weight. **c** Total number of viable head-kidney leukocytes (HKLs) per fish. **d** Respiratory burst activity. The cold experimental temperature (13 °C) and the cold host and parasite origin are indicated by the *blue* bars and the warm experimental temperature (24 °C) and the warm host and parasite origin by the *red* bars. Sample sizes are shown inside the bars and bars represent means ± SE from sham-exposed (sham), exposed but not infected (exposed) and infected sticklebacks

Laboratory reared sticklebacks from the cold and the warm origin were exposed to uninfected copepods (sham-exposed) or to copepods infected with a single *S. solidus* from either the cold or the warm origin. The infection was allowed to develop for 3 weeks, to avoid temperature effects on the infection rates, after this time half of the sticklebacks were adjusted to the cold experimental temperature and half to the warm experimental temperature. It is common that not all *S. solidus* exposed sticklebacks become infected [18, 37]. Therefore, the parasite-exposed group was further divided into ‘exposed but not infected’ and ‘infected’ sticklebacks. Together with the sham-exposed groups, this resulted in ten treatment groups per experimental temperature; the x-axis legend in Fig. 1 details all of the experimental groups.

### Parasite exposure

In total, 432 adolescent sticklebacks (216 from the cold and 216 from the warm origin) were taken from 24 families (12 families per origin) and allocated in a balanced design to experimental treatments. Nine sticklebacks per origin, 18 in total, were placed together in 14 l tanks. Sticklebacks from each origin were allocated to sham exposure and parasite exposure (cold/warm parasite origin) in a way that each of the ten exposure treatments was represented in every tank at least once. For the parasite exposure we used copepods that were singly infected with *S. solidus* larvae from 24 families (12 families per origin) in a balanced design. Experimental tanks were organized in two identical sets for the two temperature treatments.

Three weeks prior to copepod exposure, the sticklebacks were marked with Visible Implant Elastomer Tags (Northwest Marine Technologies, USA) and placed in the experimental tanks. Subsequently, all tanks were adjusted from 16 °C to 18 °C (∆*T* 0.3 °C per day). This resulted in a starting temperature that was approximately mid-way between the two experimental temperatures (13 and 24 °C) and it was also the same temperature as previous studies testing stickleback immune activity [30]. The sticklebacks were starved 72 h prior to copepod exposure and 24 h before exposure they were measured to the nearest mm (infection length) and transferred to individual glass jars with 300 ml tank water. The following day, 80 sticklebacks (40 per origin) were given one uninfected copepod to eat and 352 sticklebacks (176 per origin) were given one copepod infected with one *S. solidus* (88 *S. solidus* infected copepods per host origin/parasite origin combination). The next day water from each jar was sieved and screened to confirm ingestion of copepods and the sticklebacks were returned to their experimental tanks. To minimize temperature effects on the infection rate, exposed fish were kept at 18 °C, to give the parasites time to establish in the body cavities of the sticklebacks [37]. Thereafter, the water temperature was changed to 13 °C (∆*T* 0.8 °C per day) for half of the experimental tanks and to 24 °C (∆*T* 1 °C per day) for the other half. With the lower temperature, 13 °C, we intended to match a range that still permits the growth of hosts and parasites. By using 24 °C we aimed to challenge the hosts and parasites physiological capabilities.

### Dissection of experimental sticklebacks

The sticklebacks were dissected 56–58 days after copepod exposure when we expected that the parasites had achieved sexual maturity, i.e. exceeded the 50 mg threshold [51], at both experimental temperatures. They were anesthetised with MS-222 (Sigma-Aldrich, USA) and measured to the nearest mm (dissection length). Stickleback length increase was calculated by subtracting the infection length from the dissection length and served as a measure of stickleback fitness (health). The sticklebacks were decapitated and the body cavities were opened. To measure host immune parameters, the right head kidney was removed aseptically and transferred to 40 μm cell strainers (Falcon, BD, USA) placed in Petri dishes (ø 35 mm) with 1 ml heparinized (2 × 10^4^ IU l^-1^; Sigma-Aldrich) R-90 medium (RPMI-1640 with 10 mmol l^-1^ HEPES (both Gibco, Thermo Fisher Scientific, USA) and 10% (v/v) distilled water) on ice. The gender of the sticklebacks was recorded, the gonads were removed and the gonad weight was determined to the nearest 0.1 mg as a measure of stickleback fitness (fecundity). Subsequently, the tapeworms were removed from the infected sticklebacks and the parasite weight was determined to the nearest mg as a measure of parasite fitness [15, 16].

### Leukocyte isolation

Stickleback head kidney leukocytes (HKLs) were isolated as described in Scharsack et al. [52]. Briefly, cells and media were kept on ice, single cell suspensions of HKLs were prepared by forcing the tissues through the strainers with a syringe plunger. The HKLs were washed once with heparinized R-90 (600× *g*, 4 °C, 5 min), once with pure R-90, and resuspended in 500 μl R-90. Cell numbers were determined by means of flow cytometry and adjusted to 1.25 × 10^6^ viable HKLs ml^-1^.

### Immune assays

Sticklebacks increase their numbers of head kidney leukocytes (HKLs) and their respiratory burst activity, one of the most important effector mechanisms of cellular innate immunity, during *S. solidus* infection [18, 53]. With the present study, we wanted to test whether temperature alters the leukocyte responses to *S. solidus* infection. Total numbers of viable HKLs per fish were determined by means of flow cytometry (FACSCanto II, BD, USA) with the standard cell dilution assay [54] as described by Scharsack et al. [53]. Briefly, HKL suspensions were measured with propidium iodide (2 mg l^-1^; Sigma-Aldrich) and green fluorescent reference particles (Fluoresbrite YG Carboxylate Microspheres 4.5 μm, Polysciences, USA). Flow cytometric data were analysed with the FacsDiva software (version 6.1.2, BD). Dead cells (propidium iodide positive) and cellular debris (low light scatter characteristics) were excluded and viable HKLs were identified according to their characteristic scatter profiles.

The respiratory burst activity of HKLs was measured in a lucigenin-enhanced chemiluminescence assay [55] modified by Kurtz et al. [56]. Viable HKLs, 1 × 10^5^ per well of white 96-well microplates (Nunc, Thermo Fisher Scientific, USA), were pre-incubated with lucigenin (0.28 g l^-1^; Sigma-Aldrich) for 30 min and respiratory burst was induced by the addition of zymosan (0.75 g l^-1^; Sigma-Aldrich). Relative luminescence units (RLUs) of individual wells were measured for 3 h at 20 °C with an Infinite 200 microplate reader (Tecan, Männedorf, Switzerland). For data analyses, the area under the kinetic curve (RLU area) was determined using the Magellan 6.5 software (Tecan).

### Statistical analyses

We first tested the effect of experimental temperature (13 or 24 °C), host origin (cold or warm), parasite origin (cold or warm) and host gender (male or female) on the probability of sticklebacks becoming infected by *S. solidus* using a generalized linear model (GLM) in a binary logistic regression.

The host response variables (length increase, gonad weight, number of head-kidney leukocytes (HKLs) and respiratory burst activity) were analysed in two separate subsets since sham-exposed sticklebacks had missing values for parasite origin: (i) data from all uninfected sticklebacks (sham-exposed and exposed but not infected); and (ii) data from all parasite exposed sticklebacks (exposed but not infected and infected). After visual inspection of the response variable distributions, all response variables were Box-Cox transformed. Using linear models (LMs), we tested the effects of experimental temperature, host gender, host infection status (sham-exposed or exposed but not infected and exposed but not infected or infected) and host origin on each response variable (Additional file 1: Table S1). Parasite origin was only included in models with data from parasite-exposed sticklebacks. The response variable parasite weight was tested with the above factors, but excluding host infection status. We included all possible two- and three-way interactions in all models. Host dissection length was included as a covariate in models that depended on host size (i.e. host length increase, host gonad weight, number of HKLs and parasite weight). Sequential Bonferroni corrected *post-hoc* comparisons were computed to investigate significant interaction effects (Additional file 1: Table S2).

To test whether host tolerance varied according to our experimental treatments, we analysed two different response variables: host length increase (indicating health tolerance), and gonad weight (indicating fecundity tolerance). Using LMs, we tested whether there was an effect of experimental temperature, host origin, parasite origin or host gender on health or fecundity tolerance (Additional file 1: Table S3). Parasite weight was included as a continuous predictor variable in each model and all possible interactions were included up to three-way. A significant interaction between any of the factors and parasite weight would reveal that this factor affects host tolerance [19, 24]. Tolerance is illustrated as the slope of the relationship between host health or fecundity, and parasite weight, where steeper positive slopes indicate greater tolerance. Host dissection length was included as a covariate in both models to account for the dependency of host length increase and host gonad weight on the host dissection length. After accounting for this, the slopes were positive because larger fish generally have larger parasites. In further models we replaced host and parasite origin with a factor that described whether the combination of host and parasite was sympatric or allopatric. Where we observed significant differences in host tolerance, we compared the slopes of the regressions by sequential Bonferroni corrected, pairwise comparisons using the/LMATRIX subcommand.

We considered including the quadratic term of parasite weight into our tolerance models to account for non-linear relationships [19–21, 24, 27, 57]. Only including the linear term for parasite weight plus all other factors and interactions up to three-way, meant testing the effects of 25 parameters in total. However, adding a quadratic term for parasite weight and all subsequent interactions would have resulted in models with up to 37 parameters, which would clearly overfit our models [58] when considering that we had 204 observations, i.e. infected fish, for the tolerance models. Thus, we decided to restrict the models to linear relationships in order to avoid model-overfitting.

Whenever we found significant interaction terms in any of the models, we checked the underlying main effects and lower level interactions for validity by simple effects tests before interpretation. A Chi-square test of independence was calculated comparing the frequency of mortality in sticklebacks kept at 13 and 24 °C. All statistical analyses were performed in SPSS Statistics version 23 (IBM, USA).

## Results

### Sample size

From the 432 sticklebacks used for the experiment, 50 were excluded from data analysis: 11 for technical reasons (see Additional file 1: Table S4 for details) and 39 fish died (16 died prior to temperature change, two at 13 °C and 21 at 24 °C). The mortality was significantly higher at 24 °C compared to 13 °C (*χ* ^2^_(1,*N* = 405)_ = 15.96, *P* < 0.001). Apart from that, mortality did not depend on any of the other experimental variables (see Additional file 1: Table S5 for details). Due to low numbers of head kidney leukocytes (HKLs) an additional 38 sticklebacks could not be used in the respiratory burst assay (see Fig. 1 for detailed sample sizes).

### *Schistocephalus solidus* infection and parasite fitness

**Fig. 2:**
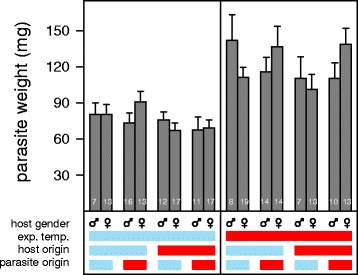
Parasite weight. The cold experimental temperature (13 °C) and the cold host and parasite origin are indicated by the *blue* bars and the warm experimental temperature (24 °C) and the warm host and parasite origin by the *red* bars. Sample sizes are shown inside the bars and bars represent means ± SE

Of the 309 sticklebacks exposed to *S. solidus* and used for data analysis, 204 became infected (mean infected: 67%; range across treatment groups: 51 to 81% infected). The infection rate did not significantly depend on any of the experimental variables. Parasites grew significantly larger in their hosts at 24 °C compared to 13 °C (*F*_(1,188)_ = 266.95, *P* < 0.001; Fig. 2) and parasite growth also depended on host gender (*F*_(1,188)_ = 7.08, *P* = 0.008), but neither parasite nor host origin had a significant influence on parasite growth (Additional file 1: Table S1).

### Host fitness

**Fig. 3:**
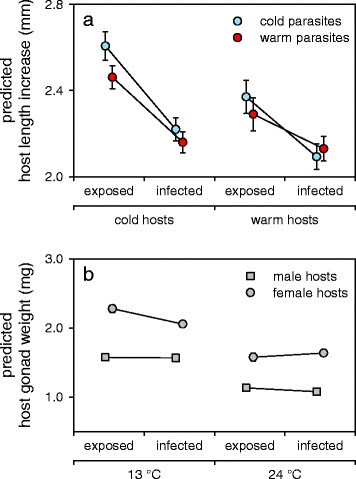
Three-way interactions explaining variation in host body condition. **a** The significant interaction (*F*_(1,282)_ = 5.40, *P* = 0.021) for host length increase between host infection status (exposed but not infected (exposed) or infected), host origin (cold or warm) and parasite origin (cold or warm). **b** The significant interaction (*F*_(1,282)_ = 9.95, *P* = 0.002) for host gonad weight between host infection status (exposed but not infected or infected), experimental temperature (13 or 24 °C) and host gender (male or female). Means ± SE (they are too small to be visible in Fig. 3b) are model-predictions from Box-Cox transformed data and not congruent with the values from Fig. 1a, b. For *P*-values of *post-hoc* comparisons between groups see Additional file 1: Table S2

To estimate stickleback fitness, we measured their length increase (Fig. 1a) and gonad weight (Fig. 1b). The sticklebacks grew faster at 13 °C compared to 24 °C (all uninfected: *F*_(1,162)_ = 158.12, *P* < 0.001 and all exposed: *F*_(1,282)_ = 265.50, *P* < 0.001). Female sticklebacks had a greater length increase than males (all exposed: *F*_(1,282)_ = 7.68, *P* = 0.006) or grew faster than males at the warm experimental temperature (all uninfected: experimental temperature × host gender, *F*_(1,162)_ = 5.25, *P* = 0.023). *Schistocephalus solidus* infection significantly decreased host growth (all exposed: *F*_(1,282)_ = 51.43, *P* < 0.001). Among *S. solidus* exposed sticklebacks, cold-origin sticklebacks grew faster than warm-origin ones (i) when exposed but not infected with parasites from the warm origin and (ii) when infected with parasites from the cold origin, which resulted in a significant three way interaction (Fig. 3a; all exposed: host infection status × host origin × parasite origin, *F*_(1,282)_ = 5.40, *P* = 0.021).

Similarly, to host growth, the sticklebacks developed larger gonads at 13 °C compared to 24 °C (all uninfected: *F*_(1,162)_ = 66.31, *P* < 0.001 and all exposed: *F*_(1,282)_ = 169.56, *P* < 0.001). The infection with *S. solidus* affected the gonad development of female but not of male sticklebacks (Additional file 1: Table S1). At 13 °C, infected female stickleback had smaller gonads than uninfected ones, but this was inverted at 24 °C resulting in higher gonad weights in infected compared to exposed but not infected females (Fig. 3b; all exposed: experimental temperature × host infection status × host gender, *F*_(1,282)_ = 9.95, *P* = 0.002). However, the effects of *S. solidus* on female gonad weight at 24 °C were only marginal compared to the prominent differences between temperature treatments.

### Host immunity

**Fig. 4:**
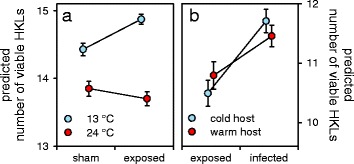
Two-way interactions explaining variation in head kidney leukocyte (HKL) numbers. **a** The significant interaction (*F*_(1,162)_ = 7.86, *P* = 0.006) between host infection status (sham-exposed (sham) or exposed but not infected (exposed)) and experimental temperature (13 or 24 °C). **b** The significant interaction (*F*_(1,282)_ = 14.32, *P* < 0.001) between host infection status (exposed but not infected or infected) and host origin (cold or warm). Means ± SE are model-predictions from Box-Cox transformed data and not congruent with the values from Fig. 1c. For *P*-values of *post-hoc* comparisons between groups see Additional file 1: Table S2

We counted the number of viable head-kidney leukocytes (HKLs; Fig. 1c) and measured their respiratory burst activity (Fig. 1d) to estimate host immune activity. Interestingly, the numbers of HKLs were increased at 13 °C in exposed but not infected sticklebacks compared to sham-exposed sticklebacks kept at the same temperature and compared to exposed but not infected sticklebacks kept at 24 °C (Fig. 4a; all uninfected: experimental temperature × host infection status, *F*_(1,162)_ = 7.86, *P* = 0.006). We collected significantly more HKLs from infected than from exposed but not infected sticklebacks (all exposed: *F*_(1,282)_ = 43.38, *P* < 0.001), although this difference was more prominent in cold-origin compared to warm-origin sticklebacks (Fig. 4b; all exposed: host infection status × host origin, *F*_(1,282)_ = 14.32, *P* < 0.001).

The respiratory burst activity was higher in HKLs from sticklebacks kept at 13 °C compared to 24 °C (all uninfected: *F*_(1,143)_ = 34.62, *P* < 0.001 and all exposed: *F*_(1,252)_ = 51.31, *P* < 0.001) and females had higher respiratory burst activity than males at 13 °C but not at 24 °C (all uninfected: experimental temperature × host gender, *F*_(1,143)_ = 5.93, *P* = 0.016 and all exposed: experimental temperature × host gender, *F*_(1,252)_ = 5.33, *P* = 0.022). The respiratory burst activity of HKLs from infected sticklebacks was strongly increased in comparison to their exposed but not infected conspecifics (all exposed: *F*_(1,252)_ = 51.87, *P* < 0.001).

### Host tolerance

**Fig. 5:**
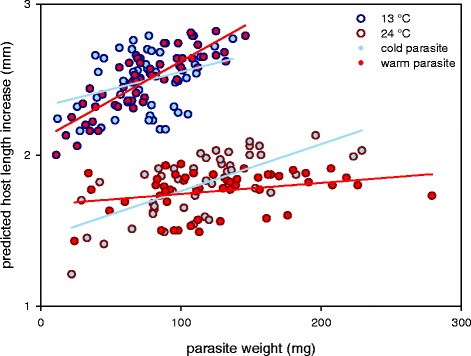
Host health tolerance. The interaction between experimental temperature (13 or 24 °C), parasite origin (cold or warm) and parasite weight (*F*_(1,177)_ = 6.72, *P* = 0.010), indicates variation in health tolerance. Steeper positive slopes of the linear relationship between host length increase and parasite weight indicate greater health tolerance. Values for host length increase are model predictions from Box-Cox transformed data and not congruent with the values from Fig. 1a

When we examined host health tolerance towards *S. solidus* infection as the relationship between host length increase and parasite weight, we observed a significant interaction between experimental temperature, parasite origin and parasite weight (Fig. 5; *F*_(1,177)_ = 6.72, *P* = 0.010). This indicates that health tolerance was differentially affected by experimental temperature and parasite origin. Sticklebacks from the cold experimental temperature, infected with *S. solidus* from the warm origin (upper red line in Fig. 5) were more tolerant when compared to (i) sticklebacks infected with cold-origin parasites at 24 °C (lower blue line; *F*_(1,196)_ = 7.36, *P* = 0.022), (ii) sticklebacks infected with cold-origin parasites at 13 °C (upper blue line; *F*_(1,196)_ = 8.14, *P* = 0.019) and (iii) the group with the lowest health tolerance, sticklebacks infected with warm-origin parasites at 24 °C (lower red line; *F*_(1,196)_ = 38.20, *P* < 0.001). Furthermore, health tolerance of sticklebacks kept at the warm experimental temperature was significantly increased when infected with parasites from the cold origin (lower blue line in Fig. 5) compared to those infected with parasites from the warm origin (lower red line; *F*_(1,196)_ = 15.43, *P* = 0.001). In contrast, fecundity tolerance measured using host gonad weight as a response variable, did not differ across treatments. Similarly, we found no effect of sympatry/allopatry on health or fecundity tolerance (Additional file 1: Table S3).

## Discussion

The warm experimental temperature used in the present study, promoted the growth of *S. solidus*, which is a correlate for parasite fecundity and fitness [15, 16]. In contrast, the ectothermic stickleback host exhibited higher fitness, measured as growth rate and gonad development, at the cooler experimental temperature. Moreover, warm conditions down-regulated stickleback immunity, i.e. the number of head kidney leukocytes (HKLs), especially in response to parasite infection, and the respiratory burst activity. Warm conditions even seemed to be detrimental to sticklebacks since host mortality was elevated at the warm experimental temperature. Altogether, these observations suggest that host fitness decreases and parasite fitness increases with rising temperature, the latter could be due to a combination of a higher parasite metabolic rate, and also because of a less efficient host immune response.

When infected with cold-origin *S. solidus*, sticklebacks from the cold origin grew faster than those from the warm origin, suggesting adaptations of the host to the local parasite genotype. However, contrary to our expectations we did not find further evidence for mutual adaptations between local host-parasite pairs but both hosts and parasites showed signs of adaptations to the temperature in their habitat of origin. This is illustrated by sticklebacks from the cold site of Lake Mývatn growing faster and showing higher plasticity in their leukocyte responses towards *S. solidus* infection than their conspecifics from the warm origin. Previous studies have revealed that experimentally cold-adapted three-spined sticklebacks had faster growing offspring compared to conspecifics that were experimentally warm adapted, illustrating that temperature might cause transgenerational plasticity [31–33]. The present study used offspring from parents collected from warm and cold environments, thus the observed temperature adaptations might be due to transgenerational effects.

Millet et al. [6] demonstrated that sticklebacks sampled at warm and cold sites of Lake Mývatn differed phenotypically, but showed little genetic variation. They suggested that Mývatn sticklebacks show relatively broad phenotypic plasticity and explained the lack of genetic divergence by the existence of gene flow across the lake and the relatively young age of the Mývatn stickleback population. Given that there is a lack of genetic divergence in the study population, signs of temperature adaptation described in the present study, might be based on transgenerational plasticity of temperature adaptation.

Temperature adaptations were also found for the parasite. *Schistocephalus solidus* was capable of exploiting its host more efficiently at its temperature of origin, suggested by lower health tolerance of infected sticklebacks at the parasite’s temperature of origin. In contrast, the health tolerance of sticklebacks was higher when infected with parasites at the parasite’s ‘foreign’ temperature. Thus the present study suggests adaptation of the parasites to their local temperature, which has costs for their hosts. However, in comparison to the differences provoked by the cold and the warm experimental temperatures, the signs of local temperature adaptation were relatively subtle, suggesting that the current environmental conditions have a greater impact on host physiology than genetic or somatic contributions from the parents. In contrast to the observed effect of parasite origin on host health tolerance, hosts from different origins did not differ in health tolerance, neither in fecundity tolerance. Similarly, we found no variation in fecundity tolerance, nor did we find an effect of sympatry/allopatry on host tolerance. In the wild, transition of parasites from their adaptive temperature to a ‘foreign’ temperature regime might result in relatively higher host tolerance, suggesting that intruding parasites cause lower host fitness reduction compared to sympatric coevolved ones. Given that the final host of *S. solidus* is a bird, there would be ample opportunities for the dispersion of the parasite. In global warming scenarios, increased host fitness costs due to predicted higher parasite life-cycle completion and dispersal rates [46], could be compensated for, at least to some extent by host tolerance. There is an increasing appreciation of the importance of abiotic factors on host tolerance [24]. Recently, it was described that *Drosophila melanogaster* infected with a fungus seek cooler temperatures, under which they were able to increase resistance to the fungal infection and increase their late life reproductive output [59]. Temperature preferences were not tested in the present study, but our data suggest that seeking of cooler temperature would also benefit sticklebacks infected with *S. solidus*, since parasites would grow less, while hosts could invest more in immunity, growth and fecundity.

Sticklebacks that were exposed to *S. solidus* but did not develop an infection at 13 °C, had increased numbers of HKLs compared to sham-exposed controls, which is intriguing since it means that an unsuccessful parasite infection left an imprint on the host immune system eight weeks after parasite exposure. The rejection of parasite larvae likely occurred during the first 2 weeks after infection [37], consequently we did not observe any parasite residuals in the host body cavity. It is possible that parasite antigens were still present in the exposed but not infected sticklebacks, which triggered the immune system, but interestingly increased numbers of HKLs in exposed but not infected sticklebacks were only observed at 13 °C, the more optimal temperature for the host.

## Conclusions

The present study demonstrates that *S. solidus* parasites benefit from warmer temperatures, whereas the fitness and immunity of their stickleback hosts are generally higher in the cold. This strongly suggests that temperature variation interferes with fitness trade-offs in host-parasite interactions, in addition to interfering with host health tolerance to *S. solidus* infection. However, both hosts and parasites were plastic in their responses to temperature and at least the plasticity of the host seems to be transgenerational mediated.

## Additional file

Additional file 1: Figure S1. Water temperature in Lake Mývatn at the cold (blue) and warm (red) sampling site from 20/06/14 to 19/06/15. **Table S1.** Linear models (LMs) on host and parasite body condition parameters and host immunity. Simple comparisons for all interpretable interactions (I1–I13) can be found in Table S2. *Symbols*: ‘-‘; not included into the model, ‘n. s.’; not significant (*P* > 0.050), ‘(…)’, not interpretable because of a significant higher level interaction effect. **Table S2.** Simple comparisons for significant, interpretable interactions (see Table S1 for an overview of all significant effects). *Symbols*: ‘n. s.’, not significant (*P* > 0.050). **Table S3.** Linear models (LMs) on host tolerance. *Symbols*: ‘-‘, not included into the model; ‘n. s.’, not significant (*P* > 0.050). **Table S4.** Technical reasons for excluding 11 sticklebacks from data analysis. **Table S5.** Distribution of dead sticklebacks over experimental treatments. ‘Count’ gives the number of dead sticklebacks in the order males/females/unknown gender. *Symbols*: ‘?’, host infection status unknown. (PDF 131 kb)

## Abbreviations

FACS: Fluorescence accelerated cell scanner
GLM: Generalized linear model
HEPES: 2-[4-(2-hydroxyethyl) piperazin-1-yl] ethanesulfonic acid
HKL: Head kidney leukocyte
LM: Linear model
MEM: Minimal essential medium
MS-222: Ethyl 3-aminobenzoate methanesulfonate
R-90: RPMI-1640 with 10 mmol l^-1^ HEPES and 10% v/v distilled water
RLU: Relative luminescence unit
RPMI-1640: Roswell Park Memorial Institute cell culture medium no. 1640
YG: yellow, green
