# Supplementary material for: Environmental temperature variation influences fitness trade-offs and tolerance in a fish-tapeworm association
Source: Parasit Vectors. 2017 Jun 2;10:252. doi: 10.1186/s13071-017-2192-7 (PMC5455083; doi:10.1186/s13071-017-2192-7)
Supplement: Additional file 1: Figure S1. — Water temperature in Lake Mývatn at the cold (blue) and warm (red) sampling site from 20/06/14 to 19/06/15. Table S1. Linear models (LMs) on host and parasite body condition parameters and host immunity. Simple comparisons for all interpretable interactions (I1–I13) can be found in Table S2. Symbols: ‘-‘; not included into the model, ‘n. s.’; not significant (P > 0.050), ‘(…)’, not interpretable because of a significant higher level interaction effect. Table S2. Simple comparisons for significant, interpretable interactions (see Table S1 for an overview of all significant effects). Symbols: ‘n. s.’, not significant (P > 0.050). Table S3. Linear models (LMs) on host tolerance. Symbols: ‘-‘, not included into the model; ‘n. s.’, not significant (P > 0.050). Table S4. Technical reasons for excluding 11 sticklebacks from data analysis. Table S5. Distribution of dead sticklebacks over experimental treatments. ‘Count’ gives the number of dead sticklebacks in the order males/females/unknown gender. Symbols: ‘?’, host infection status unknown. (PDF 131 kb) [file 13071_2017_2192_MOESM1_ESM.pdf]

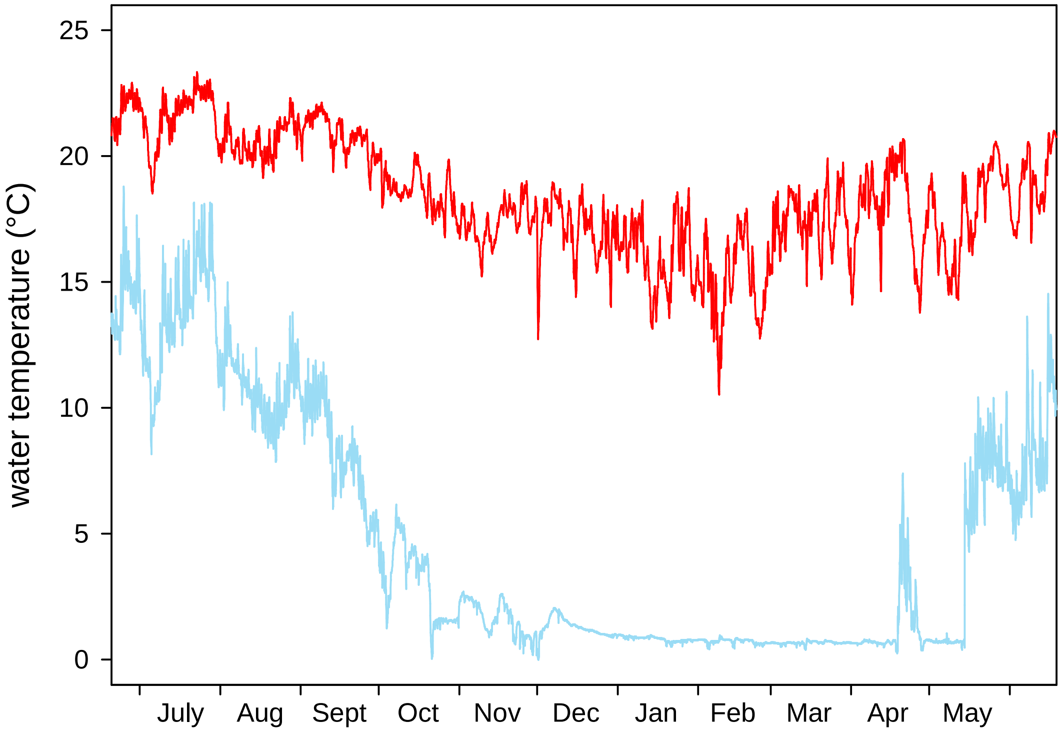

Figure S1. Water temperature in Lake Mývatn at the cold (blue) and warm (red) sampling site from 20/06/14 to 19/06/15.

Table S1. Linear models (LMs) on host and parasite body condition parameters and host immunity. Simple comparisons for all interpretable interactions (I1 - I13) can be found in Table S2. *Symbols*: ‘-‘; not included into the model, ‘n. s.’; not significant (P > 0.050), ‘(…)’, not interpretable because of a significant higher level interaction effect.

	models include all uninfected sticklebacks (sham-exposed and exposed but not infected)				model includes all parasite exposed sticklebacks (exposed but not infected and infected)				
	host length increase	host gonad weight	number of viable HKLs	respiratory burst activity	host length increase	host gonad weight	number of viable HKLs	respiratory burst activity	parasite weight
experimental temperature	$F_{1,162} = 158.12$, $P < 0.001$	$F_{1,162} = 66.31$, $P < 0.001$	($F_{1,162} = 23.48$, $P < 0.001$)	$F_{1,143} = 34.62$, $P < 0.001$	$F_{1,282} = 265.50$, $P < 0.001$	$F_{1,282} = 169.56$, $P < 0.001$	($F_{1,282} = 140.65$, $P < 0.001$)	$F_{1,252} = 51.31$, $P < 0.001$	$F_{1,188} = 266.95$, $P < 0.001$
host gender	($F_{1,162} = 4.82$, $P = 0.030$)	$F_{1,162} = 75.30$, $P < 0.001$	n. s.	($F_{1,143} = 5.14$, $P = 0.025$)	$F_{1,282} = 7.68$, $P = 0.006$	$F_{1,282} = 206.63$, $P < 0.001$	n. s.	($F_{1,252} = 9.54$, $P = 0.002$)	$F_{1,188} = 7.08$, $P = 0.008$
host infection status	n. s.	n. s.	n. s.	n. s.	$F_{1,282} = 51.43$, $P < 0.001$	n. s.	($F_{1,282} = 43.38$, $P < 0.001$)	$F_{1,252} = 51.87$, $P < 0.001$	-
host origin	n. s.	n. s.	($F_{1,162} = 5.77$, $P = 0.017$)	n. s.	($F_{1,282} = 11.55$, $P = 0.001$)	n. s.	n. s.	n. s.	n. s.
parasite origin	-	-	-	-	n. s.	n. s.	n. s.	n. s.	n. s.
experimental temperature × host gender	I1: $F_{1,162} = 5.25$, $P = 0.023$	n. s.	I2: $F_{1,162} = 8.90$, $P = 0.003$	I5: $F_{1,143} = 5.93$, $P = 0.016$	n. s.	I7: $F_{1,282} = 9.53$, $P = 0.002$	($F_{1,282} = 6.11$, $P = 0.014$)	I12: $F_{1,252} = 5.33$, $P = 0,022$	n. s.
experimental temperature × host infection status	n. s.	n. s.	I3: $F_{1,162} = 7.86$, $P = 0.006$	n. s.	n. s.	n. s.	($F_{1,282} = 10.36$, $P = 0.001$)	n. s.	-
experimental temperature × host origin	n. s.	n. s.	I4: $F_{1,162} = 4.04$, $P = 0.046$	n. s.	n. s.	n. s.	n. s.	n. s.	n. s.
experimental temperature × parasite origin	-	-	-	-	n. s.	n. s.	n. s.	n. s.	n. s.
host gender × host infection status	n. s.	n. s.	n. s.	n. s.	n. s.	n. s.	n. s.	n. s.	-
host gender × host origin	n. s.	n. s.	n. s.	n. s.	n. s.	n. s.	n. s.	n. s.	n. s.
host gender × parasite origin	-	-	-	-	n. s.	n. s.	n. s.	n. s.	n. s.
host infection status × host origin	n. s.	n. s.	n. s.	n. s.	n. s.	n. s.	I10: $F_{1,282} = 14.32$, $P < 0.001$	n. s.	-
host infection status × parasite origin	-	-	-	-	n. s.	n. s.	n. s.	n. s.	-
host origin × parasite origin	-	-	-	-	n. s.	n. s.	n. s.	n. s.	n. s.
experimental temperature × host gender × host infection status	n. s.	n. s.	n. s.	n. s.	n. s.	I8: $F_{1,282} = 9.953$, $P = 0.002$	I11: $F_{1,282} = 4.740$, $P = 0.030$	n. s.	-
experimental temperature × host gender × host origin	n. s.	n. s.	n. s.	n. s.	n. s.	n. s.	n. s.	n. s.	n. s.
experimental temperature × host gender × parasite origin	-	-	-	-	n. s.	n. s.	n. s.	n. s.	n. s.
experimental temperature × host infection status × host origin	n. s.	n. s.	n. s.	n. s.	n. s.	n. s.	n. s.	n. s.	-
experimental temperature × host infection status × parasite origin	-	-	-	-	n. s.	n. s.	n. s.	n. s.	-
experimental temperature × host origin × parasite origin	-	-	-	-	n. s.	n. s.	n. s.	n. s.	n. s.
host gender × host infection status × host origin	n. s.	n. s.	n. s.	n. s.	n. s.	n. s.	n. s.	I13: $F_{1,252} = 4.06$, $P = 0.045$	-
host gender × host infection status × parasite origin	-	-	-	-	n. s.	n. s.	n. s.	n. s.	-
host gender × host origin × parasite origin	-	-	-	-	n. s.	n. s.	n. s.	n. s.	n. s.
host infection status × host origin × parasite origin	-	-	-	-	I6: $F_{1,282} = 5.40$, $P = 0.021$	I9: $F_{1,282} = 4.25$, $P = 0.040$	n. s.	n. s.	-

Table S2. Simple comparisons for significant, interpretable interactions (see Table S1 for an overview of all significant effects). *Symbols*: ‘n. s.’, not significant (P > 0.050).

interaction (see table S1)	1st interaction effect	2nd interaction effect	3rd interaction effect	*P*-value
I1	**exp. temperature**	**host gender**	-	
model on host length increase	13 °C	male vs. female		n. s.
exp. temperature × host gender	24 °C	male vs. female		0.002
$F_{1,162}$ = 5.25, *P* = 0.023	**host gender**	**exp. temperature**	-	
	male	13 °C vs. 24 °C		< 0.001
	female	13 °C vs. 24 °C		< 0.001
I2	**exp. temperature**	**host gender**	-	
model on number of viable HKLs	13 °C	male vs. female		0.043
exp. temperature × host gender	24 °C	male vs. female		0.038
$F_{1,162}$ = 8.90, *P* = 0.003	**host gender**	**exp. temperature**	-	
	male	13 °C vs. 24 °C		< 0.001
	female	13 °C vs. 24 °C		n. s.
I3	**exp. temperature**	**host infection status**	-	
model on number of viable HKLs	13 °C	sham vs. exposed		0.014
exp. temperature × host infection status	24 °C	sham vs. exposed		n. s.
$F_{1,162}$ = 7.86, *P* = 0.006	**host infection status**	**exp. temperature**	-	
	sham	13 °C vs. 24 °C		n. s.
	exposed	13 °C vs. 24 °C		< 0.001
I4	**exp. temperature**	**host origin**	-	
model on number of viable HKLs	13 °C	cold vs. warm		n. s.
exp. temperature × host origin	24 °C	cold vs. warm		0.002
$F_{1,162}$ = 4.04, *P* = 0.046	**host origin**	**exp. temperature**	-	
	cold	13 °C vs. 24 °C		< 0.001
	warm	13 °C vs. 24 °C		0.045
I5	**exp. temperature**	**host gender**	-	
model on respiratory burst activity	13 °C	male vs. female		< 0.001
exp. temperature × host gender	24 °C	male vs. female		n. s.
$F_{1,143}$ = 5.93, *P* = 0.016	**host gender**	**exp. temperature**	-	
	male	13 °C vs. 24 °C		0.023
	female	13 °C vs. 24 °C		< 0.001
I6	**host infection ststus**	**host origin**	**parasite origin**	
model on host length increase	exposed	cold	cold vs. warm	n. s.
host infection status × host origin × parasite origin	exposed	warm	cold vs. warm	n. s.
$F_{1,282}$ = 5.40, *P* = 0.021	infected	cold	cold vs. warm	n. s.
	infected	warm	cold vs. warm	n. s.
	**host infection statsus**	**parasite origin**	**host origin**	
	exposed	cold	cold vs. warm	n. s.
	exposed	warm	cold vs. warm	0.020
	infected	cold	cold vs. warm	0.001
	infected	warm	cold vs. warm	n. s.
	**host origin**	**parasite origin**	**host infection statsus**	
	cold	cold	exposed vs. infected	0.014
	cold	warm	exposed vs. infected	< 0.001
	warm	cold	exposed vs. infected	< 0.001
	warm	warm	exposed vs. infected	0.020

interaction (see table S1)	1st interaction effect	2nd interaction effect	3rd interaction effect	*P*-value
I7	**exp. temperature**	**host gender**	-	
model on host gonad weight	13 °C	male vs. female		< 0.001
exp. temperature × host gender	24 °C	male vs. female		< 0.001
$F_{1,282}$ = 9.53, *P* = 0.002	**host gender**	**exp. temperature**	-	
	male	13 °C vs. 24 °C		< 0.001
	female	13 °C vs. 24 °C		< 0.001
I8	**exp. temperature**	**host gender**	**host infection ststus**	
model on host gonad weight	13 °C	male	exposed vs. infected	n. s.
exp. temperature × host gender × host infection status	13 °C	female	exposed vs. infected	0.008
$F_{1,282}$ = 9.95, *P* = 0.002	24 °C	male	exposed vs. infected	n. s.
	24 °C	female	exposed vs. infected	0.016
	**exp. temperature**	**host infection status**	**host gender**	
	13 °C	exposed	male vs. female	< 0.001
	13 °C	infected	male vs. female	< 0.001
	24 °C	exposed	male vs. female	< 0.001
	24 °C	infected	male vs. female	< 0.001
	**host gender**	**host infection status**	**exp. temperature**	
	male	exposed	13 °C vs 24 °C	< 0.001
	male	infected	13 °C vs 24 °C	< 0.001
	female	exposed	13 °C vs 24 °C	< 0.001
	female	infected	13 °C vs 24 °C	< 0.001
I9	**host infection ststus**	**host origin**	**parasite origin**	
model on host gonad weight	exposed	cold	cold vs. warm	n. s.
host infection status × host origin × parasite origin	exposed	warm	cold vs. warm	n. s.
$F_{1,282}$ = 4.25, *P* = 0.040	infected	cold	cold vs. warm	n. s.
	infected	warm	cold vs. warm	n. s.
	**host infection statsus**	**parasite origin**	**host origin**	
	exposed	cold	cold vs. warm	n. s.
	exposed	warm	cold vs. warm	n. s.
	infected	cold	cold vs. warm	0.047
	infected	warm	cold vs. warm	n. s.
	**host origin**	**parasite origin**	**host infection statsus**	
	cold	cold	exposed vs. infected	0.022
	cold	warm	exposed vs. infected	n. s.
	warm	cold	exposed vs. infected	n. s.
	warm	warm	exposed vs. infected	n. s.
I10	**host infection status**	**host origin**	-	
model on number of viable HKLs	exposed	cold vs. warm		0.001
host infection status × host origin	infected	cold vs. warm		n. s.
$F_{1,282}$ = 14.32, *P* < 0.001	**host origin**	**host infection ststus**	-	
	cold	exposed vs. infected		< 0.001
	warm	exposed vs. infected		0.050

Interaction (see table S1)	1st interaction effect	2nd interaction effect	3rd interaction effect	*P*-value
**I11**	**exp. temperature**	**host gender**	**host infection ststus**	
model on number of viable HKLs	13 °C	**male**	exposed vs. infected	< 0.001
exp. temperature × host gender × host infection status	13 °C	female	exposed vs. infected	< 0.001
$F_{1,282}$ = 4.74, P = 0.030	24 °C	male	exposed vs. infected	0.005
	24 °C	female	exposed vs. infected	n. s.
	**exp. temperature**	**host infection status**	**host gender**	
	13 °C	exposed	male vs. female	0.020
	13 °C	infected	male vs. female	n. s.
	24 °C	exposed	male vs. female	n. s.
	24 °C	infected	male vs. female	n. s.
	**host gender**	**host infection status**	**exp. temperature**	
	male	exposed	13 °C vs 24 °C	< 0.001
	male	infected	13 °C vs 24 °C	< 0.001
	female	exposed	13 °C vs 24 °C	n. s.
	female	infected	13 °C vs 24 °C	< 0.001
**I12**	**exp. temperature**	**host gender**	**-**	
model on respiratory burst activity	13 °C	male vs. female		< 0.001
exp. temperature × host gender	24 °C	male vs. female		n. s.
$F_{1,252}$ = 5.33, P = 0,022	**host gender**	**exp. temperature**	**-**	
	male	13 °C vs. 24 °C		0.002
	female	13 °C vs. 24 °C		< 0.001
**I13**	**host gender**	**host infection status**	**host origin**	
model on respiratory burst activity	male	exposed	cold vs. warm	n. s.
host gender × host infection status × host origin	male	infected	cold vs. warm	n. s.
$F_{1,252}$ = 4.06, P = 0.045	female	exposed	cold vs. warm	0.008
	female	infected	cold vs. warm	n. s.
	**host gender**	**host origin**	**host infection status**	
	male	cold	exposed vs. infected	< 0.001
	male	warm	exposed vs. infected	0.001
	female	cold	exposed vs. infected	0.034
	female	warm	exposed vs. infected	< 0.001
	**host infection status**	**host origin**	**host gender**	
	exposed	cold	male vs. female	0.001
	exposed	warm	male vs. female	n. s.
	infected	cold	male vs. female	n. s.
	infected	warm	male vs. female	n. s.

Table S3. Linear models (LMs) on host tolerance. *Symbols*: '-', not included into the model; 'n. s.', not significant (P > 0.050).

	including host/parasite origins		including sympatry/allopatry	
	host health tolerance	host fecundity tolerance	host health tolerance	host fecundity tolerance
experimental temperature	$F = {}_{1,177} = 25.35$, $P < 0.001$	$F_{1,177} = 10.71$, $P = 0.001$	$F_{1,188} = 21.09$, $P < 0.001$	$F_{1,188} = 10.19$, $P = 0.002$
host gender	n. s.	$F_{1,177} = 18.92$, $P < 0.001$	$F_{1,188} = 4.11$, $P = 0.044$	$F_{1,188} = 29.70$, $P < 0.001$
host origin	n. s.	n. s.	-	-
parasite origin	n. s.	n. s.	-	-
parasite weight	n. s.	n. s.	n. s.	n. s.
sympatry/allopatry	-	-	n. s.	n. s.
experimental temperature × host gender	n. s.	n. s.	n. s.	n. s.
experimental temperature × host origin	n. s.	n. s.	-	-
experimental temperature × parasite origin	$F_{1,177} = 5.97$, $P = 0.015$	n. s.	-	-
experimental temperature × parasite weight	n. s.	n. s.	n. s.	n. s.
experimental temperature × sympatry/allopatry	-	-	n. s.	n. s.
host gender × host origin	n. s.	n. s.	-	-
host gender × parasite origin	n. s.	n. s.	-	-
host gender × parasite weight	n. s.	n. s.	n. s.	n. s.
host gender × sympatry/allopatry	-	-	n. s.	n. s.
host origin × parasite origin	n. s.	n. s.	-	-
host origin × parasite weight	n. s.	n. s.	-	-
parasite origin × parasite weight	n. s.	n. s.	-	-
parasite weight × sympatry/allopatry	-	-	n. s.	n. s.
experimental temperature × host gender × host origin	n. s.	n. s.	-	-
experimental temperature × host gender × parasite origin	n. s.	n. s.	-	-
experimental temperature × host gender × parasite weight	n. s.	n. s.	n. s.	n. s.
experimental temperature × host gender × sympatry/allopatry	-	-	n. s.	n. s.
experimental temperature × host origin × parasite origin	n. s.	n. s.	-	-
experimental temperature × host origin × parasite weight	n. s.	n. s.	-	-
experimental temperature × parasite origin × parasite weight	$F_{1,177} = 6.72$, $P = 0.010$	n. s.	-	-
experimental temperature × parasite weight × sympatry/allopatry	-	-	n. s.	n. s.
host gender × host origin × parasite origin	n. s.	n. s.	-	-
host gender × host origin × parasite weight	n. s.	n. s.	-	-
host gender × parasite origin × parasite weight	n. s.	n. s.	-	-
host gender × parasite weight × sympatry/allopatry	-	-	n. s.	n. s.
host origin × parasite origin × parasite weight	n. s.	n. s.	-	-

Table S4. Technical reasons for excluding 11 sticklebacks from data analysis.

count	technical reason
2	stickleback infected with two *S. solidus* plerocercoids
1	stickleback with deformed tail
2	gender of the stickleback could not be determined
1	kidney was extremely swollen
3	stickleback disappeared
1	stickleback did not eat the copepod
1	sham exposed stickleback was *S. solidus* infected

Table S5. Distribution of dead sticklebacks over experimental treatments. 'Count' gives the number of dead sticklebacks in the order males/females/unknown gender. *Symbols*: '?', host infection status unknown.

Experimental temperature (°C)	host infection status	host origin	parasite origin	count
18	sham exposed	cold	-	-/-/5
18	sham exposed	warm	-	-/-/6
18	?	cold	cold	-/-/1
18	?	cold	warm	-/-/1
18	?	warm	cold	-/-/2
18	?	warm	warm	-/-/1
13	sham exposed	cold	-	-/1/-
13	infected	cold	warm	-/-/1
24	exposed but not infected	warm	cold	1/-/-
24	sham exposed	cold	-	-/-/1
24	sham exposed	warm	-	-/-/1
24	exposed but not infected	cold	cold	-/1/-
24	exposed but not infected	warm	cold	2/-/1
24	exposed but not infected	warm	warm	-/-/1
24	infected	cold	cold	1/1/2
24	infected	warm	cold	2/2/-
24	infected	warm	warm	2/1/1
24	?	warm	warm	-/-/1
